# Trimethyl­ammonium 5-(2,4-dinitro­phenyl)-2,6-dioxo-1,2,3,6-tetra­hydro­pyrimidin-4-olate 0.125-hydrate

**DOI:** 10.1107/S1600536813007915

**Published:** 2013-03-28

**Authors:** Govindan Mangaiyarkarasi, Doraisamyraja Kalaivani

**Affiliations:** a PG and Research Department of Chemistry, Seethalakshmi Ramaswami College, Tiruchirappalli 620 002, Tamil Nadu, India

## Abstract

The asymmetric unit of the title salt C_3_H_10_N^+^·C_10_H_5_N_4_O_7_^−^·0.125H_2_O [trivial name: trimethyl­ammonium 5-(2,4-dinitro­phen­yl)barbiturate 0.125-hydrate], contains two independent cations, two independent anions and a 0.25-occupancy solvent water mol­ecule. In one of the cations, the C atoms are disordered over two sets of sites with refined occupancies of 0.538 (8) and 0.462 (8). In the anions, the dihedral angles between the pyrimidine and benzene rings are 42.77 (6) and 46.55 (7)°. In the crystal, N—H⋯O hydrogen bonds connect anions and cations into chains along [010]. Within these chains, *R*_2_^2^(8) ring motifs are formed by inversion-related barbiturate anions. The H atoms of the partial occupancy water mol­ecule were not located nor included in the refinement.

## Related literature

For the different types of anionic sigma complexes, see: Terrier (1982 ▶); Al-Kaysi *et al.* (2005 ▶). For the utility of spiro Meisenheimer complexes, see: Gallardo *et al.* (2007 ▶); Al-Kaysi *et al.* 2008 ▶). For the biological activity of carbanionic sigma complexes related to the title compound, see: Kalaivani *et al.* (2008 ▶); Kalaivani & Buvaneswari (2010 ▶). For the crystal structures of related barbiturates, see: Kalaivani & Malarvizhi (2009 ▶); Kalaivani *et al.* (2012 ▶). For hydrogen-bond graph-set motifs, see: Bernstein *et al.* (1995 ▶).
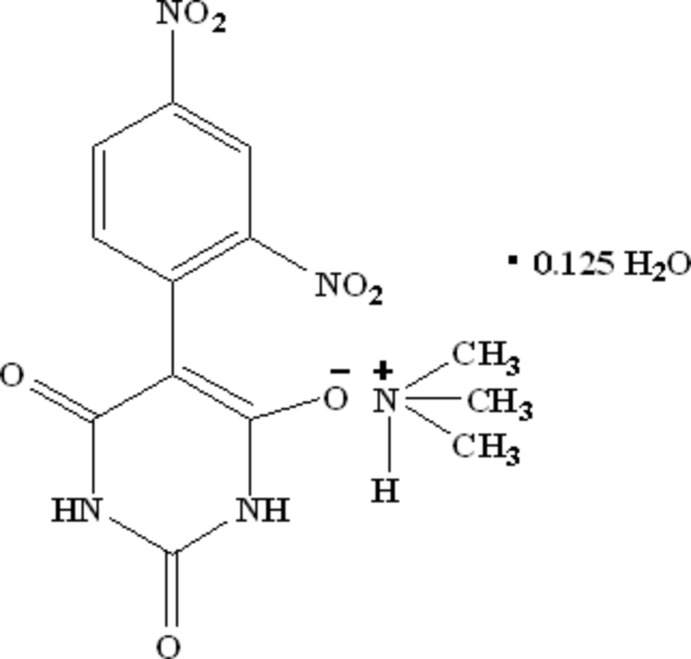


## Experimental

### 

#### Crystal data

C_3_H_10_N^+^·C_10_H_5_N_4_O_7_^−^·0.125H_2_O
*M* *_r_* = 355.29
Monoclinic, 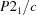

*a* = 15.0410 (5) Å
*b* = 10.5460 (3) Å
*c* = 20.4170 (8) Å
β = 94.953 (1)°
*V* = 3226.50 (19) Å^3^
*Z* = 8
Mo *K*α radiation
μ = 0.12 mm^−1^
*T* = 293 K
0.30 × 0.20 × 0.20 mm

#### Data collection

Bruker Kappa APEXII CCD diffractometer
Absorption correction: multi-scan (*SADABS*; Bruker, 2004 ▶) *T*_min_ = 0.960, *T*_max_ = 0.987
26803 measured reflections
5517 independent reflections
3780 reflections with *I* > 2σ(*I*)
*R*_int_ = 0.039

#### Refinement

*R*[*F* ^2^ > 2σ(*F* ^2^)] = 0.045
*wR*(*F* ^2^) = 0.130
*S* = 1.02
5517 reflections
516 parameters
36 restraints
H atoms treated by a mixture of independent and constrained refinement
Δρ_max_ = 0.21 e Å^−3^
Δρ_min_ = −0.21 e Å^−3^

### 

Data collection: *APEX2* (Bruker, 2004 ▶); cell refinement: *SAINT* (Bruker, 2004 ▶); data reduction: *SAINT*; program(s) used to solve structure: *SIR92* (Altomare *et al.*, 1993 ▶); program(s) used to refine structure: *SHELXL97* (Sheldrick, 2008 ▶); molecular graphics: *ORTEP-3 for Windows* (Farrugia, 2012 ▶) and *Mercury* (Macrae *et al.*, 2006) ▶; software used to prepare material for publication: *SHELXL97*.

## Supplementary Material

Crystal structure: contains datablock(s) global, I. DOI: 10.1107/S1600536813007915/lh5595sup1.cif

Structure factors: contains datablock(s) I. DOI: 10.1107/S1600536813007915/lh5595Isup2.hkl

Supplementary material file. DOI: 10.1107/S1600536813007915/lh5595Isup3.cml

Additional supplementary materials:  crystallographic information; 3D view; checkCIF report

## Figures and Tables

**Table 1:** Hydrogen-bond geometry (Å, °)

*D*—H⋯*A*	*D*—H	H⋯*A*	*D*⋯*A*	*D*—H⋯*A*
N1—H1*A*⋯O9^i^	0.80 (2)	2.09 (2)	2.888 (2)	175 (2)
N2—H2*A*⋯O8^ii^	0.89 (3)	2.02 (3)	2.905 (2)	173 (2)
N5—H5*A*⋯O3^i^	0.84 (2)	2.10 (2)	2.931 (2)	169 (2)
N6—H6*A*⋯O1^ii^	0.85 (2)	2.04 (3)	2.889 (2)	177 (2)
N9—H9*A*⋯O10	1.04 (3)	1.62 (4)	2.650 (2)	167 (3)
N10—H10⋯O2	1.02 (4)	1.63 (4)	2.644 (3)	173 (3)

Symmetry codes: (i) 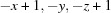; (ii) 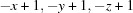.

**Fig. 1.**
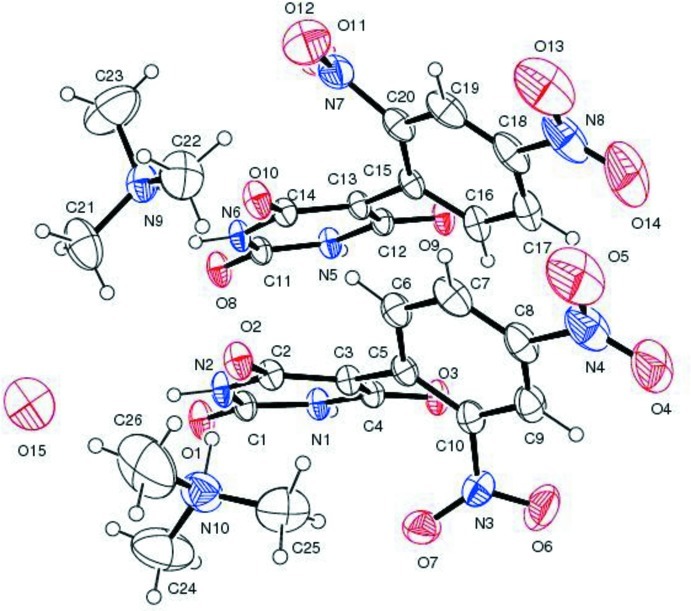
The asymmetric unit of the title compound, with displacement ellipsoids drawn at the 30% probability level.

**Fig. 2.**
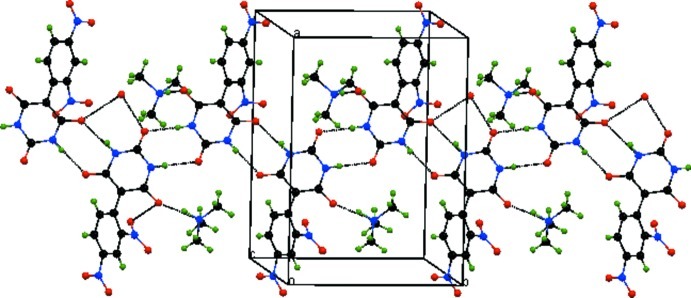
Part of the crystal atructure of the title compound with hydrogen bonds shown as dashed lines.

## supplementary crystallographic information

### Comment

Many different types of anionic sigma complexes such as carbon bonded, nitrogen bonded, oxygen bonded and spiro Meisenheimer complexes have been reported by different group of scientists (Terrier, 1982; Al-Kaysi *et al.*, 2005). Among these, spiro Meisenheimer complexes have notable photophysical and redox properties which have enabled their utility in the field of electro chemistry (Gallardo *et al.*, 2007; Al-Kaysi *et al.*,2008). In our laboratories, we have synthesized new carbanionic sigma complexes from aromatic nitro compounds (1-Chloro-2,4-dinitrobenzene and 1-chloro-2,4,6-trinitrobenzene) and the ketone [pyrimidine-2,4,6(1H,3H,5H)-trione (barbituric acid)] in the presence of tertiary amines which have noticable anticonvulsant/hypnotic activities (Kalaivani & Malarvizhi, 2009; Kalaivani & Buvaneswari, 2010). In this article, we report another new type of carbanionic sigma complex which is a pyrimidine derivative (barbiturate), isolated from the ethanolic solutions of 1-chloro-2,4-dinitrobenzene, barbituric acid and trimethylamine.

The asymmetric unit of the title compund (Fig. 1) comprises of two cations and two anions. In addition, the asymmetric unit contains a 0.25 occupancy water molecule. The bond lengths and bond angles of the title compound are comparible with those of related barbiturates (Kalaivani & Malarvizhi, 2009; Kalaivani *et al.*, 2012). In one the cations the C atoms C24, C25 and C26 are disordered over two sets of sites with refined occupancies of 0.538 (8) and 0.462 (8). Since there are two nitro groups attached to the benzene rings in the anions a steric effect may operate which is reflected in the dihedral angles between the benzene and pyrimidine rings (42.77 (6) and 46.55 (7)°). The nitro group para with respect to barbiturate ring are approximately planar (8.2 (4) and 6.0 (5)°) with benzene ring and are effectively involved in the delocalisation of electrons. The nitro groups ortho with respect to barbiturate ring form dihedral angles of 40.7 (3) and 33.8 (3)° with the benzene rings. In the crystal, N—H···O hydrogen bonds connect anions and cations into one-dimensional chains along [010]. Within these chains *R*_2_^2^(8)ring motifs (Bernstein *et al.*, 1995) are formed by inversion-related barbiturate anions (Fig. 2).

### Experimental

1-Chloro-2,4-dinitrobenzene (2.02 g, 0.01 mol) was dissolved in 20 ml of absolute alcohol. Barbituric acid (1.28 g, 0.01 mol) was also dissolved in 30 ml of absolute alcohol. After mixing these two solutions, 3 ml of trimethylamine (0.03 mol) was added and shaken well for 6 hrs. The solution was filtered and the clear solution was kept as such at room temperature. After a period of four weeks, dark shiny maroon red coloured crystals formed from the solution. The crystals were filtered and washed with 30 ml of dry ether and recrystallized from absolute ethanol (M.p: 545K; yield: 80%). Good quality crystals for single crystal X-ray studies were obtained by slow evaporation of ethanol at room temperature.

### Refinement

The N-bound H atoms were located in a difference electron density map and refind with a N—H distance restraint of 0.90 (2) Å. The C-bound hydrogen atoms were placed in calculated positions and refined as riding atoms: C—H = 0.93 and 0.96 Å for CH and CH_3_ H atoms, respectively, with *U*_iso_(H) = k × *U*_eq_(C), where k = 1.5 for methyl H atoms and = 1.2 for other H atoms. The H atoms of the partial occupancy water molecule were not located nor are they included in the refinement. They are however included in the molecular formula.

### Crystal data

**Table:**

C_3_H_10_N^+^·C_10_H_5_N_4_O_7_^−^·0.125H_2_O	*F*(000) = 1482
*M**_r_* = 355.29	*D*_x_ = 1.464 Mg m^−^^3^
Monoclinic, *P*2_1_/*c*	Mo *K*α radiation, λ = 0.71073 Å
Hall symbol: -P 2ybc	Cell parameters from 7398 reflections
*a* = 15.0410 (5) Å	θ = 2.3–23.9°
*b* = 10.5460 (3) Å	µ = 0.12 mm^−^^1^
*c* = 20.4170 (8) Å	*T* = 293 K
β = 94.953 (1)°	Needle, red
*V* = 3226.50 (19) Å^3^	0.30 × 0.20 × 0.20 mm
*Z* = 8	

### Data collection

**Table:**

Bruker Kappa APEXII CCD diffractometer	5517 independent reflections
Radiation source: fine-focus sealed tube	3780 reflections with *I* > 2σ(*I*)
Graphite monochromator	*R*_int_ = 0.039
ω and φ scans	θ_max_ = 24.7°, θ_min_ = 2.2°
Absorption correction: multi-scan (*SADABS*; Bruker, 2004)	*h* = −17→17
*T*_min_ = 0.960, *T*_max_ = 0.987	*k* = −12→12
26803 measured reflections	*l* = −24→23

### Refinement

**Table:**

Refinement on *F*^2^	Secondary atom site location: difference Fourier map
Least-squares matrix: full	Hydrogen site location: inferred from neighbouring sites
*R*[*F*^2^ > 2σ(*F*^2^)] = 0.045	H atoms treated by a mixture of independent and constrained refinement
*wR*(*F*^2^) = 0.130	*w* = 1/[σ^2^(*F*_o_^2^) + (0.0564*P*)^2^ + 1.2781*P*] where *P* = (*F*_o_^2^ + 2*F*_c_^2^)/3
*S* = 1.02	(Δ/σ)_max_ < 0.001
5517 reflections	Δρ_max_ = 0.21 e Å^−^^3^
516 parameters	Δρ_min_ = −0.21 e Å^−^^3^
36 restraints	Extinction correction: *SHELXL97* (Sheldrick, 2008), Fc^*^=kFc[1+0.001xFc^2^λ^3^/sin(2θ)]^-1/4^
Primary atom site location: structure-invariant direct methods	Extinction coefficient: 0.0014 (4)

### Special details

**Table:**

Geometry. All esds (except the esd in the dihedral angle between two l.s. planes) are estimated using the full covariance matrix. The cell esds are taken into account individually in the estimation of esds in distances, angles and torsion angles; correlations between esds in cell parameters are only used when they are defined by crystal symmetry. An approximate (isotropic) treatment of cell esds is used for estimating esds involving l.s. planes.
Refinement. Refinement of F^2^ against ALL reflections. The weighted R-factor wR and goodness of fit S are based on F^2^, conventional R-factors R are based on F, with F set to zero for negative F^2^. The threshold expression of F^2^ > 2sigma(F^2^) is used only for calculating R-factors(gt) etc. and is not relevant to the choice of reflections for refinement. R-factors based on F^2^ are statistically about twice as large as those based on F, and R- factors based on ALL data will be even larger.

### Fractional atomic coordinates and isotropic or equivalent isotropic displacement parameters (Å^2^)

**Table:**

	*x*	*y*	*z*	*U*_iso_*/*U*_eq_	Occ. (<1)
C1	0.49436 (14)	0.3608 (2)	0.41756 (12)	0.0407 (5)	
C2	0.34407 (14)	0.3929 (2)	0.36471 (11)	0.0397 (5)	
C3	0.33767 (13)	0.26371 (19)	0.34916 (11)	0.0375 (5)	
C4	0.40782 (14)	0.18045 (19)	0.37118 (11)	0.0373 (5)	
C5	0.25811 (14)	0.2167 (2)	0.31023 (11)	0.0412 (5)	
C6	0.17278 (15)	0.2572 (2)	0.32317 (13)	0.0508 (6)	
H6	0.1672	0.3167	0.3562	0.061*	
C7	0.09634 (16)	0.2114 (3)	0.28831 (15)	0.0630 (8)	
H7	0.0406	0.2431	0.2962	0.076*	
C8	0.10415 (18)	0.1187 (3)	0.24204 (14)	0.0611 (8)	
C9	0.18518 (19)	0.0769 (3)	0.22658 (13)	0.0603 (7)	
H9	0.1895	0.0146	0.1947	0.072*	
C10	0.26101 (15)	0.1286 (2)	0.25901 (12)	0.0472 (6)	
C11	0.48116 (14)	0.24816 (19)	0.58140 (11)	0.0399 (5)	
C12	0.38713 (14)	0.06518 (19)	0.54816 (11)	0.0382 (5)	
C13	0.31760 (13)	0.14908 (19)	0.52559 (11)	0.0376 (5)	
C14	0.33028 (13)	0.28091 (19)	0.53044 (11)	0.0389 (5)	
C15	0.23348 (14)	0.1023 (2)	0.49394 (12)	0.0422 (6)	
C16	0.23265 (17)	0.0110 (2)	0.44466 (13)	0.0525 (6)	
H16	0.2862	−0.0268	0.4359	0.063*	
C17	0.1550 (2)	−0.0257 (2)	0.40826 (14)	0.0646 (8)	
H17	0.1562	−0.0876	0.3759	0.077*	
C18	0.07617 (18)	0.0312 (3)	0.42094 (16)	0.0675 (9)	
C19	0.07232 (17)	0.1193 (3)	0.46899 (16)	0.0670 (8)	
H19	0.0183	0.1569	0.4769	0.080*	
C20	0.14985 (15)	0.1520 (2)	0.50569 (14)	0.0530 (7)	
C21	0.2649 (2)	0.6911 (3)	0.4683 (2)	0.0951 (12)	
H21A	0.2330	0.7699	0.4647	0.143*	
H21B	0.2811	0.6660	0.4257	0.143*	
H21C	0.3179	0.7012	0.4976	0.143*	
C22	0.1287 (2)	0.5725 (3)	0.44899 (19)	0.0935 (11)	
H22A	0.0955	0.5024	0.4643	0.140*	
H22B	0.1462	0.5540	0.4059	0.140*	
H22C	0.0921	0.6473	0.4472	0.140*	
C23	0.1874 (4)	0.6194 (5)	0.5611 (2)	0.1438 (19)	
H23A	0.1611	0.7020	0.5632	0.216*	
H23B	0.2412	0.6162	0.5901	0.216*	
H23C	0.1462	0.5567	0.5743	0.216*	
N1	0.48201 (12)	0.23460 (17)	0.40610 (10)	0.0400 (5)	
N2	0.42297 (12)	0.43476 (18)	0.39760 (10)	0.0450 (5)	
N3	0.34457 (16)	0.0974 (2)	0.23077 (11)	0.0627 (6)	
N4	0.0238 (2)	0.0651 (3)	0.20626 (16)	0.0905 (9)	
N5	0.46542 (12)	0.12161 (16)	0.57581 (10)	0.0411 (5)	
N6	0.41223 (12)	0.32321 (17)	0.55772 (10)	0.0412 (5)	
N7	0.13846 (15)	0.2384 (2)	0.56078 (15)	0.0695 (7)	
N8	−0.0066 (2)	−0.0018 (4)	0.38078 (18)	0.0998 (11)	
N9	0.20830 (14)	0.5938 (2)	0.49399 (12)	0.0585 (6)	
N10	0.31143 (19)	0.5901 (3)	0.23666 (15)	0.0822 (8)	
C24	0.3960 (6)	0.6339 (15)	0.2289 (5)	0.139 (4)	0.538 (8)
H24A	0.4118	0.6979	0.2612	0.208*	0.538 (8)
H24B	0.3975	0.6694	0.1857	0.208*	0.538 (8)
H24C	0.4376	0.5650	0.2343	0.208*	0.538 (8)
C25	0.2718 (9)	0.4906 (11)	0.1921 (8)	0.100 (3)	0.538 (8)
H25A	0.2127	0.4713	0.2035	0.149*	0.538 (8)
H25B	0.3080	0.4156	0.1963	0.149*	0.538 (8)
H25C	0.2690	0.5204	0.1475	0.149*	0.538 (8)
C26	0.2437 (8)	0.7042 (9)	0.2306 (6)	0.166 (5)	0.538 (8)
H26A	0.1848	0.6738	0.2367	0.249*	0.538 (8)
H26B	0.2439	0.7417	0.1878	0.249*	0.538 (8)
H26C	0.2609	0.7665	0.2635	0.249*	0.538 (8)
C24'	0.4071 (5)	0.5293 (11)	0.2295 (5)	0.099 (3)	0.462 (8)
H24D	0.4483	0.5583	0.2649	0.149*	0.462 (8)
H24E	0.4278	0.5545	0.1883	0.149*	0.462 (8)
H24F	0.4027	0.4386	0.2311	0.149*	0.462 (8)
C25'	0.2503 (10)	0.5448 (16)	0.1872 (11)	0.133 (6)	0.462 (8)
H25D	0.2447	0.4546	0.1913	0.200*	0.462 (8)
H25E	0.2707	0.5648	0.1451	0.200*	0.462 (8)
H25F	0.1933	0.5839	0.1909	0.200*	0.462 (8)
C26'	0.3305 (12)	0.7243 (9)	0.2384 (7)	0.158 (5)	0.462 (8)
H26D	0.3714	0.7431	0.2758	0.238*	0.462 (8)
H26E	0.2761	0.7708	0.2414	0.238*	0.462 (8)
H26F	0.3567	0.7484	0.1989	0.238*	0.462 (8)
O1	0.56366 (10)	0.40504 (14)	0.44398 (9)	0.0549 (5)	
O2	0.28668 (10)	0.47693 (14)	0.34922 (9)	0.0503 (4)	
O3	0.40882 (10)	0.06395 (13)	0.36237 (8)	0.0474 (4)	
O4	0.03283 (18)	−0.0250 (3)	0.16961 (14)	0.1164 (10)	
O5	−0.04734 (18)	0.1135 (3)	0.21523 (16)	0.1303 (11)	
O6	0.35400 (17)	−0.0101 (2)	0.21072 (13)	0.1034 (8)	
O7	0.39882 (13)	0.1810 (2)	0.22575 (10)	0.0735 (6)	
O8	0.55192 (10)	0.29224 (14)	0.60553 (9)	0.0517 (4)	
O9	0.38360 (10)	−0.05263 (13)	0.54731 (9)	0.0501 (4)	
O10	0.27374 (10)	0.36162 (14)	0.51066 (9)	0.0544 (5)	
O11	0.18612 (14)	0.2261 (2)	0.61110 (12)	0.0802 (6)	
O12	0.07827 (14)	0.3173 (2)	0.55353 (15)	0.1107 (9)	
O13	−0.07427 (19)	0.0598 (3)	0.39067 (16)	0.1369 (13)	
O14	−0.0034 (2)	−0.0854 (4)	0.34104 (18)	0.1460 (14)	
O15	0.3059 (7)	0.8311 (10)	0.3338 (6)	0.112 (3)	0.25
H1A	0.5215 (16)	0.188 (2)	0.4194 (12)	0.047 (7)*	
H2A	0.4307 (17)	0.519 (3)	0.4003 (13)	0.065 (8)*	
H5A	0.5069 (16)	0.075 (2)	0.5918 (12)	0.048 (7)*	
H6A	0.4201 (15)	0.403 (2)	0.5586 (11)	0.046 (7)*	
H9A	0.242 (2)	0.507 (3)	0.4982 (16)	0.100 (10)*	
H10	0.301 (2)	0.553 (3)	0.2815 (19)	0.101 (11)*	

### Atomic displacement parameters (Å^2^)

**Table:**

	*U*^11^	*U*^22^	*U*^33^	*U*^12^	*U*^13^	*U*^23^
C1	0.0379 (12)	0.0329 (11)	0.0502 (15)	0.0000 (10)	−0.0031 (11)	0.0029 (10)
C2	0.0346 (12)	0.0361 (11)	0.0472 (14)	0.0022 (10)	−0.0031 (10)	0.0048 (10)
C3	0.0345 (11)	0.0343 (11)	0.0425 (13)	0.0005 (9)	−0.0043 (10)	0.0018 (10)
C4	0.0378 (12)	0.0331 (11)	0.0405 (13)	−0.0002 (9)	0.0015 (10)	0.0010 (10)
C5	0.0413 (13)	0.0330 (11)	0.0482 (14)	−0.0021 (9)	−0.0025 (11)	0.0100 (10)
C6	0.0417 (14)	0.0448 (13)	0.0650 (17)	−0.0020 (11)	−0.0008 (12)	0.0049 (12)
C7	0.0403 (14)	0.0642 (17)	0.082 (2)	−0.0041 (12)	−0.0072 (14)	0.0227 (16)
C8	0.0547 (17)	0.0672 (18)	0.0574 (18)	−0.0231 (14)	−0.0187 (14)	0.0147 (15)
C9	0.0689 (19)	0.0590 (16)	0.0505 (17)	−0.0171 (14)	−0.0097 (14)	0.0023 (13)
C10	0.0492 (14)	0.0428 (13)	0.0478 (15)	−0.0063 (11)	−0.0054 (12)	0.0035 (11)
C11	0.0346 (12)	0.0325 (11)	0.0520 (15)	−0.0001 (9)	−0.0004 (11)	0.0023 (10)
C12	0.0351 (12)	0.0315 (11)	0.0473 (14)	0.0007 (9)	0.0004 (10)	0.0007 (10)
C13	0.0302 (11)	0.0312 (11)	0.0503 (14)	−0.0010 (9)	−0.0020 (10)	0.0023 (10)
C14	0.0323 (12)	0.0327 (11)	0.0509 (14)	0.0025 (9)	−0.0005 (10)	0.0037 (10)
C15	0.0361 (12)	0.0334 (11)	0.0557 (15)	−0.0035 (9)	−0.0049 (11)	0.0116 (11)
C16	0.0523 (15)	0.0396 (13)	0.0634 (17)	−0.0073 (11)	−0.0073 (13)	0.0049 (12)
C17	0.077 (2)	0.0494 (15)	0.0633 (19)	−0.0224 (14)	−0.0185 (15)	0.0129 (13)
C18	0.0473 (17)	0.076 (2)	0.075 (2)	−0.0262 (15)	−0.0223 (15)	0.0277 (17)
C19	0.0365 (14)	0.078 (2)	0.085 (2)	−0.0084 (13)	−0.0068 (14)	0.0288 (18)
C20	0.0357 (13)	0.0511 (14)	0.0710 (18)	−0.0039 (11)	−0.0030 (12)	0.0156 (13)
C21	0.073 (2)	0.0631 (19)	0.146 (3)	−0.0026 (16)	−0.014 (2)	0.024 (2)
C22	0.0570 (19)	0.099 (3)	0.121 (3)	0.0029 (17)	−0.0150 (19)	−0.006 (2)
C23	0.202 (5)	0.153 (4)	0.079 (3)	0.050 (4)	0.025 (3)	−0.009 (3)
N1	0.0356 (10)	0.0291 (10)	0.0535 (13)	0.0048 (8)	−0.0074 (9)	0.0019 (9)
N2	0.0393 (11)	0.0268 (10)	0.0666 (14)	0.0017 (8)	−0.0092 (10)	−0.0007 (9)
N3	0.0672 (15)	0.0638 (15)	0.0561 (15)	0.0007 (13)	0.0006 (12)	−0.0126 (12)
N4	0.072 (2)	0.116 (3)	0.078 (2)	−0.0380 (19)	−0.0195 (16)	0.0198 (19)
N5	0.0321 (10)	0.0303 (10)	0.0591 (13)	0.0031 (8)	−0.0073 (9)	0.0012 (9)
N6	0.0375 (10)	0.0248 (9)	0.0595 (13)	−0.0011 (8)	−0.0055 (9)	0.0018 (9)
N7	0.0423 (13)	0.0651 (15)	0.103 (2)	0.0020 (12)	0.0183 (14)	−0.0020 (15)
N8	0.073 (2)	0.120 (3)	0.098 (3)	−0.047 (2)	−0.0348 (19)	0.046 (2)
N9	0.0573 (13)	0.0423 (12)	0.0743 (17)	0.0121 (10)	−0.0035 (12)	0.0044 (11)
N10	0.093 (2)	0.0820 (19)	0.0712 (19)	−0.0091 (16)	0.0058 (16)	0.0140 (16)
C24	0.078 (5)	0.220 (12)	0.120 (6)	−0.044 (7)	0.019 (5)	0.010 (9)
C25	0.077 (7)	0.147 (9)	0.071 (5)	−0.001 (6)	−0.016 (5)	0.007 (6)
C26	0.163 (9)	0.097 (6)	0.240 (11)	0.042 (6)	0.033 (9)	0.079 (7)
C24'	0.070 (5)	0.145 (9)	0.085 (6)	−0.017 (6)	0.016 (4)	0.036 (7)
C25'	0.070 (8)	0.214 (17)	0.107 (8)	−0.013 (9)	−0.041 (6)	0.016 (12)
C26'	0.199 (12)	0.099 (7)	0.168 (9)	−0.004 (8)	−0.043 (10)	0.033 (7)
O1	0.0434 (9)	0.0375 (8)	0.0794 (13)	−0.0030 (7)	−0.0200 (9)	0.0010 (8)
O2	0.0409 (9)	0.0354 (8)	0.0724 (12)	0.0064 (7)	−0.0071 (8)	0.0059 (8)
O3	0.0463 (9)	0.0297 (8)	0.0637 (11)	0.0029 (7)	−0.0088 (8)	−0.0038 (7)
O4	0.113 (2)	0.138 (2)	0.093 (2)	−0.0637 (18)	−0.0212 (16)	−0.0162 (18)
O5	0.0599 (15)	0.183 (3)	0.142 (3)	−0.0275 (18)	−0.0307 (16)	0.010 (2)
O6	0.1144 (19)	0.0792 (16)	0.119 (2)	0.0062 (14)	0.0223 (16)	−0.0437 (15)
O7	0.0633 (12)	0.0905 (15)	0.0681 (14)	−0.0123 (11)	0.0136 (10)	−0.0077 (11)
O8	0.0375 (9)	0.0384 (8)	0.0757 (12)	−0.0049 (7)	−0.0147 (8)	0.0018 (8)
O9	0.0439 (9)	0.0267 (8)	0.0772 (12)	0.0019 (6)	−0.0098 (8)	−0.0011 (8)
O10	0.0398 (9)	0.0344 (8)	0.0866 (13)	0.0058 (7)	−0.0094 (8)	0.0104 (8)
O11	0.0713 (14)	0.0872 (15)	0.0830 (16)	−0.0027 (11)	0.0122 (12)	−0.0153 (13)
O12	0.0624 (13)	0.1029 (18)	0.169 (3)	0.0362 (13)	0.0214 (15)	−0.0064 (17)
O13	0.0581 (16)	0.193 (3)	0.151 (3)	−0.0261 (19)	−0.0423 (17)	0.038 (2)
O14	0.135 (3)	0.155 (3)	0.135 (3)	−0.078 (2)	−0.064 (2)	0.006 (2)
O15	0.096 (7)	0.117 (8)	0.120 (9)	0.001 (6)	−0.012 (6)	0.015 (7)

### Geometric parameters (Å, º)

**Table:**

C1—O1	1.224 (2)	C22—N9	1.462 (4)
C1—N2	1.361 (3)	C22—H22A	0.9600
C1—N1	1.361 (3)	C22—H22B	0.9600
C2—O2	1.258 (2)	C22—H22C	0.9600
C2—N2	1.384 (3)	C23—N9	1.458 (5)
C2—C3	1.401 (3)	C23—H23A	0.9600
C3—C4	1.416 (3)	C23—H23B	0.9600
C3—C5	1.465 (3)	C23—H23C	0.9600
C4—O3	1.242 (2)	N1—H1A	0.80 (2)
C4—N1	1.394 (3)	N2—H2A	0.89 (3)
C5—C6	1.399 (3)	N3—O7	1.212 (3)
C5—C10	1.402 (3)	N3—O6	1.217 (3)
C6—C7	1.386 (3)	N4—O5	1.213 (4)
C6—H6	0.9300	N4—O4	1.225 (4)
C7—C8	1.372 (4)	N5—H5A	0.84 (2)
C7—H7	0.9300	N6—H6A	0.85 (2)
C8—C9	1.359 (4)	N7—O11	1.208 (3)
C8—N4	1.470 (4)	N7—O12	1.229 (3)
C9—C10	1.381 (3)	N8—O14	1.202 (5)
C9—H9	0.9300	N8—O13	1.239 (5)
C10—N3	1.465 (3)	N9—H9A	1.04 (3)
C11—O8	1.225 (2)	N10—C24	1.375 (8)
C11—N5	1.359 (3)	N10—C25'	1.390 (12)
C11—N6	1.360 (3)	N10—C26'	1.445 (9)
C12—O9	1.244 (2)	N10—C25	1.480 (10)
C12—N5	1.394 (3)	N10—C26	1.575 (8)
C12—C13	1.416 (3)	N10—C24'	1.593 (9)
C13—C14	1.406 (3)	N10—H10	1.02 (4)
C13—C15	1.456 (3)	C24—H24A	0.9600
C14—O10	1.246 (2)	C24—H24B	0.9600
C14—N6	1.382 (3)	C24—H24C	0.9600
C15—C16	1.392 (3)	C25—H25A	0.9600
C15—C20	1.402 (3)	C25—H25B	0.9600
C16—C17	1.384 (3)	C25—H25C	0.9600
C16—H16	0.9300	C26—H26A	0.9600
C17—C18	1.374 (4)	C26—H26B	0.9600
C17—H17	0.9300	C26—H26C	0.9600
C18—C19	1.356 (4)	C24'—H24D	0.9600
C18—N8	1.471 (4)	C24'—H24E	0.9600
C19—C20	1.374 (3)	C24'—H24F	0.9600
C19—H19	0.9300	C25'—H25D	0.9600
C20—N7	1.469 (4)	C25'—H25E	0.9600
C21—N9	1.459 (4)	C25'—H25F	0.9600
C21—H21A	0.9600	C26'—H26D	0.9600
C21—H21B	0.9600	C26'—H26E	0.9600
C21—H21C	0.9600	C26'—H26F	0.9600
			
O1—C1—N2	122.3 (2)	C2—N2—H2A	116.5 (16)
O1—C1—N1	123.1 (2)	O7—N3—O6	123.4 (3)
N2—C1—N1	114.60 (19)	O7—N3—C10	118.5 (2)
O2—C2—N2	116.01 (19)	O6—N3—C10	118.1 (2)
O2—C2—C3	126.6 (2)	O5—N4—O4	124.4 (3)
N2—C2—C3	117.34 (18)	O5—N4—C8	117.5 (4)
C2—C3—C4	119.78 (18)	O4—N4—C8	118.2 (3)
C2—C3—C5	119.30 (18)	C11—N5—C12	126.03 (19)
C4—C3—C5	120.92 (18)	C11—N5—H5A	115.3 (16)
O3—C4—N1	117.40 (18)	C12—N5—H5A	118.6 (16)
O3—C4—C3	125.84 (19)	C11—N6—C14	125.57 (18)
N1—C4—C3	116.76 (18)	C11—N6—H6A	118.0 (15)
C6—C5—C10	115.5 (2)	C14—N6—H6A	116.5 (15)
C6—C5—C3	120.9 (2)	O11—N7—O12	123.5 (3)
C10—C5—C3	123.5 (2)	O11—N7—C20	119.0 (2)
C7—C6—C5	122.1 (3)	O12—N7—C20	117.5 (3)
C7—C6—H6	119.0	O14—N8—O13	125.1 (3)
C5—C6—H6	119.0	O14—N8—C18	117.9 (4)
C8—C7—C6	119.0 (3)	O13—N8—C18	116.9 (4)
C8—C7—H7	120.5	C23—N9—C21	112.8 (3)
C6—C7—H7	120.5	C23—N9—C22	112.8 (3)
C9—C8—C7	121.6 (2)	C21—N9—C22	110.7 (3)
C9—C8—N4	118.4 (3)	C23—N9—H9A	103.1 (18)
C7—C8—N4	120.0 (3)	C21—N9—H9A	110.7 (18)
C8—C9—C10	118.7 (3)	C22—N9—H9A	106.3 (18)
C8—C9—H9	120.6	C24—N10—C25'	126.3 (12)
C10—C9—H9	120.6	C24—N10—C26'	59.3 (6)
C9—C10—C5	122.8 (2)	C25'—N10—C26'	118.1 (8)
C9—C10—N3	115.1 (2)	C24—N10—C25	119.6 (8)
C5—C10—N3	121.7 (2)	C25'—N10—C25	26.4 (8)
O8—C11—N5	123.06 (19)	C26'—N10—C25	141.1 (8)
O8—C11—N6	122.11 (19)	C24—N10—C26	109.4 (7)
N5—C11—N6	114.83 (19)	C25'—N10—C26	79.7 (8)
O9—C12—N5	117.75 (18)	C26'—N10—C26	51.6 (6)
O9—C12—C13	126.16 (19)	C25—N10—C26	105.6 (6)
N5—C12—C13	116.05 (18)	C24—N10—C24'	43.4 (6)
C14—C13—C12	120.20 (18)	C25'—N10—C24'	109.9 (9)
C14—C13—C15	118.26 (18)	C26'—N10—C24'	102.6 (7)
C12—C13—C15	121.46 (18)	C25—N10—C24'	88.7 (7)
O10—C14—N6	118.06 (18)	C26—N10—C24'	152.1 (5)
O10—C14—C13	124.63 (19)	C24—N10—H10	116.9 (19)
N6—C14—C13	117.29 (18)	C25'—N10—H10	112 (2)
C16—C15—C20	115.5 (2)	C26'—N10—H10	114 (2)
C16—C15—C13	120.5 (2)	C25—N10—H10	101 (2)
C20—C15—C13	123.8 (2)	C26—N10—H10	102.1 (19)
C17—C16—C15	122.4 (3)	C24'—N10—H10	98.3 (19)
C17—C16—H16	118.8	N10—C24—H24A	109.5
C15—C16—H16	118.8	N10—C24—H24B	109.5
C18—C17—C16	118.6 (3)	H24A—C24—H24B	109.5
C18—C17—H17	120.7	N10—C24—H24C	109.5
C16—C17—H17	120.7	H24A—C24—H24C	109.5
C19—C18—C17	121.8 (2)	H24B—C24—H24C	109.5
C19—C18—N8	118.6 (3)	N10—C25—H25A	109.5
C17—C18—N8	119.6 (4)	N10—C25—H25B	109.5
C18—C19—C20	118.6 (3)	H25A—C25—H25B	109.5
C18—C19—H19	120.7	N10—C25—H25C	109.5
C20—C19—H19	120.7	H25A—C25—H25C	109.5
C19—C20—C15	123.0 (3)	H25B—C25—H25C	109.5
C19—C20—N7	115.0 (2)	N10—C26—H26A	109.5
C15—C20—N7	121.9 (2)	N10—C26—H26B	109.5
N9—C21—H21A	109.5	H26A—C26—H26B	109.5
N9—C21—H21B	109.5	N10—C26—H26C	109.5
H21A—C21—H21B	109.5	H26A—C26—H26C	109.5
N9—C21—H21C	109.5	H26B—C26—H26C	109.5
H21A—C21—H21C	109.5	N10—C24'—H24D	109.5
H21B—C21—H21C	109.5	N10—C24'—H24E	109.5
N9—C22—H22A	109.5	H24D—C24'—H24E	109.5
N9—C22—H22B	109.5	N10—C24'—H24F	109.5
H22A—C22—H22B	109.5	H24D—C24'—H24F	109.5
N9—C22—H22C	109.5	H24E—C24'—H24F	109.5
H22A—C22—H22C	109.5	N10—C25'—H25D	109.5
H22B—C22—H22C	109.5	N10—C25'—H25E	109.5
N9—C23—H23A	109.5	H25D—C25'—H25E	109.5
N9—C23—H23B	109.5	N10—C25'—H25F	109.5
H23A—C23—H23B	109.5	H25D—C25'—H25F	109.5
N9—C23—H23C	109.5	H25E—C25'—H25F	109.5
H23A—C23—H23C	109.5	N10—C26'—H26D	109.5
H23B—C23—H23C	109.5	N10—C26'—H26E	109.5
C1—N1—C4	125.44 (19)	H26D—C26'—H26E	109.5
C1—N1—H1A	117.2 (17)	N10—C26'—H26F	109.5
C4—N1—H1A	117.3 (17)	H26D—C26'—H26F	109.5
C1—N2—C2	125.73 (19)	H26E—C26'—H26F	109.5
C1—N2—H2A	117.0 (17)		
			
O2—C2—C3—C4	−179.0 (2)	C16—C17—C18—N8	177.1 (2)
N2—C2—C3—C4	4.4 (3)	C17—C18—C19—C20	0.1 (4)
O2—C2—C3—C5	1.0 (4)	N8—C18—C19—C20	−178.7 (2)
N2—C2—C3—C5	−175.6 (2)	C18—C19—C20—C15	2.8 (4)
C2—C3—C4—O3	177.7 (2)	C18—C19—C20—N7	−174.2 (2)
C5—C3—C4—O3	−2.3 (4)	C16—C15—C20—C19	−3.7 (4)
C2—C3—C4—N1	−2.0 (3)	C13—C15—C20—C19	170.6 (2)
C5—C3—C4—N1	178.0 (2)	C16—C15—C20—N7	173.1 (2)
C2—C3—C5—C6	−43.2 (3)	C13—C15—C20—N7	−12.6 (4)
C4—C3—C5—C6	136.7 (2)	O1—C1—N1—C4	−174.0 (2)
C2—C3—C5—C10	137.7 (2)	N2—C1—N1—C4	6.4 (3)
C4—C3—C5—C10	−42.4 (3)	O3—C4—N1—C1	176.6 (2)
C10—C5—C6—C7	1.0 (3)	C3—C4—N1—C1	−3.7 (3)
C3—C5—C6—C7	−178.1 (2)	O1—C1—N2—C2	176.8 (2)
C5—C6—C7—C8	3.5 (4)	N1—C1—N2—C2	−3.6 (3)
C6—C7—C8—C9	−4.4 (4)	O2—C2—N2—C1	−178.5 (2)
C6—C7—C8—N4	177.6 (2)	C3—C2—N2—C1	−1.6 (4)
C7—C8—C9—C10	0.7 (4)	C9—C10—N3—O7	136.7 (2)
N4—C8—C9—C10	178.7 (2)	C5—C10—N3—O7	−36.0 (3)
C8—C9—C10—C5	4.2 (4)	C9—C10—N3—O6	−40.0 (3)
C8—C9—C10—N3	−168.4 (2)	C5—C10—N3—O6	147.4 (3)
C6—C5—C10—C9	−4.9 (3)	C9—C8—N4—O5	−171.8 (3)
C3—C5—C10—C9	174.2 (2)	C7—C8—N4—O5	6.2 (4)
C6—C5—C10—N3	167.2 (2)	C9—C8—N4—O4	8.3 (4)
C3—C5—C10—N3	−13.7 (3)	C7—C8—N4—O4	−173.7 (3)
O9—C12—C13—C14	179.0 (2)	O8—C11—N5—C12	−179.1 (2)
N5—C12—C13—C14	1.3 (3)	N6—C11—N5—C12	0.6 (3)
O9—C12—C13—C15	−4.2 (4)	O9—C12—N5—C11	−179.6 (2)
N5—C12—C13—C15	178.1 (2)	C13—C12—N5—C11	−1.7 (3)
C12—C13—C14—O10	178.4 (2)	O8—C11—N6—C14	−179.3 (2)
C15—C13—C14—O10	1.5 (4)	N5—C11—N6—C14	1.0 (3)
C12—C13—C14—N6	0.0 (3)	O10—C14—N6—C11	−179.8 (2)
C15—C13—C14—N6	−176.9 (2)	C13—C14—N6—C11	−1.3 (3)
C14—C13—C15—C16	130.0 (2)	C19—C20—N7—O11	144.5 (3)
C12—C13—C15—C16	−46.9 (3)	C15—C20—N7—O11	−32.6 (4)
C14—C13—C15—C20	−44.1 (3)	C19—C20—N7—O12	−33.2 (3)
C12—C13—C15—C20	139.1 (2)	C15—C20—N7—O12	149.8 (2)
C20—C15—C16—C17	1.9 (3)	C19—C18—N8—O14	−175.6 (3)
C13—C15—C16—C17	−172.6 (2)	C17—C18—N8—O14	5.6 (4)
C15—C16—C17—C18	0.6 (4)	C19—C18—N8—O13	4.6 (4)
C16—C17—C18—C19	−1.7 (4)	C17—C18—N8—O13	−174.2 (3)

### Hydrogen-bond geometry (Å, º)

**Table:**

*D*—H···*A*	*D*—H	H···*A*	*D*···*A*	*D*—H···*A*
N1—H1*A*···O9^i^	0.80 (2)	2.09 (2)	2.888 (2)	175 (2)
N2—H2*A*···O8^ii^	0.89 (3)	2.02 (3)	2.905 (2)	173 (2)
N5—H5*A*···O3^i^	0.84 (2)	2.10 (2)	2.931 (2)	169 (2)
N6—H6*A*···O1^ii^	0.85 (2)	2.04 (3)	2.889 (2)	177 (2)
N9—H9*A*···O10	1.04 (3)	1.62 (4)	2.650 (2)	167 (3)
N10—H10···O2	1.02 (4)	1.63 (4)	2.644 (3)	173 (3)

Symmetry codes: (i) −*x*+1, −*y*, −*z*+1; (ii) −*x*+1, −*y*+1, −*z*+1.
